# Supplementary material for: Factors associated with quality of life (QOL) scores among methadone patients in Myanmar
Source: PLOS Glob Public Health. 2022 Aug 22;2(8):e0000469. doi: 10.1371/journal.pgph.0000469 (PMC10021231; doi:10.1371/journal.pgph.0000469)
Supplement: Attachment — Submitted filename: Response to Reviewers PLoS QOL 27.4.2022.docx [file pgph.0000469.s002.docx]

PGPH-D-21-01098
Factors affecting the quality of life (QOL) scores among methadone patients in Myanmar
PLOS Global Public Health

Dear Dr. Tun,

Thank you for submitting your manuscript to PLOS Global Public Health. After careful consideration, we feel that it has merit but does not fully meet PLOS Global Public Health’s publication criteria as it currently stands. Therefore, we invite you to submit a revised version of the manuscript that addresses the points raised during the review process.

==============================
EDITOR'S COMMENTS:

- The reviewers have assessed the strengths and weaknesses of the manuscript and made recommendation of a revision. Kindly consider the recommendations for revision they have made.

Answer: I appreciated the editor and reviewers’ insightful comments and suggestions. I addressed on these points and hereby resubmitted with revised version.

- The title require minor modification to align with the study design. Suggested modification is “Factors associated with quality-of-life scores among methadone patients in Myanmar."

Answer: Thanks for your comprehensive review and guidance. I hereby revised the title as suggested “Factors associated with quality of life (QOL) scores among methadone patients in Myanmar”.

- The study has not sufficiently operationalized quality of life measures. This requires theoretical and conceptual clarification in the introduction section.

Answer: Well noted and integrated the operationalized quality of life measures as follows, which integrated into the introduction session.

- Quality of Life is a multidimensional concept which incorporates the individual’s perception of themselves and other aspect of life. WHO developed a quality of life assessment, WHOQOL for a genuinely international measure of quality of life for continued promotion of a holistic approach to health and health care (WHO, 1999).
- To assess the general QOL relating to the patient satisfaction of the drug users, WHOQOL-100 and WHOQOL-BREF were mostly used in the studies of patients treated for substance abuse and addiction (Campêlo, Barbosa, & Porto, 2015).
- The use of WHOQOL-BREF, a short-form version of WHOQOL-100 quality of life assessment in this analysis covered an individual’s overall perception of quality of life in a conceptual coherence and health, as well as four domain scores on the person’s physical health, psychological health, social relationships and their relationship to salient features of the environment (WHO, 1999). The WHOQOL-BREF was the most commonly used test for patients treated with methadone for its reliability and validity (Pedrero-Pérez & Group, 2018). Patients with opioid use disorder had a poor QOL due to their suffering from other chronic diseases (Fei, Yee, Habil, & Danaee, 2016). However, those patients with a better QOL were associated with successful methadone treatment outcomes (Wang, et al., 2012).

Data collection and measures should be separated. Instrument development and measures should be a sub-heading describing what constitutes the variables in the instrument being measured and how these are linked with the explanatory variables and the outcome variable of quality of life.

Answer:

#### Data Collection and Instrument development and measures were separated with different titles.

- Data collection was added with “These five cities were from five States and Regions where methadone dispensing sites existed in the country. Myanmar had 55 methadone sites (including 15 one-stop-shop sites). Among these sites, sites selection was done with stratified random sampling from eligible cities, of States and Regions (Program, 2019).”
- Instrument development and measures was added with the following explanations.

“The WHOQOL-BREF instrument: an abbreviated version of the WHOQOL-100, which covered 26 questions in four domains: physical, psychological, social relationships and environment. Responses to these 26 questions were recorded on 5-point Likert scale and transformed each raw scale score to a 0-100 scale using the formula.

These are the steps for checking and cleaning data and computing domain scores for the WHOQOL-BREF.

Step 1: Check all 26 items from the assessment have a range of 1-5

Step 2: Convert 3 negatively phrased items into relevant positively framed questions.

Step 3: Compute domain scores from relevant domain questions

Step 4: Transform scores to a 0-100 scale

This step involves transforming each raw scale score to a 0-100 scale using the formula shown below:

$Transformed Scale=[\frac{(Actual raw score-lowest possible raw score)}{Possible raw score range}$] × 100

where “Actual raw score” is the value achieved through summation, and “lowest possible raw score” is the lowest possible value that could occur through summation. “Possible raw score range” is the difference between the maximum possible raw score and the lowest possible raw score (WHO, WHOQOL-BREF, 1997). These calculated QOL scores according to different domains and in-total scores were analysed with response characteristics to identify the association with QOL scores.”

- A statement about construct, content and item validity is required. Similarly, a statement of assurance of reliability or standardization of the instrument is equally essential.
- Answer: Thank you for your guidance and expand the item validity explanations are added. The validated questionnaires were translated from English to Burmese by two trained bilingual translators and translated back from Burmese to English to reflect the proper understanding and meaning of the original questions. Added as “Translated questionnaires were re-tested and noted a clear understanding of the responses. As the limited reviewers validated the translation of questionnaires, the Cronbach's Alpha coefficient was not calculated. There was no statistical validation to compare the comparability of language and similarity of interpretability.”
- Data collection procedure should follow describing how participants provided their responses, including urine samples.

Answer: more clarification of the data and urine collection procedures were added under “Ethical measure” as per guidance.

- Respondents' personal identifiable information was not elicited to mitigate the participation risk. Respondents were informed that all collected urine specimens would be immediately disposed of and their urine drug results would not be disclosed to anyone. The survey procedure was conducted in private rooms of the harm reduction drop-in-centres and private rooms of the restaurant where the respondents did not feel any external threat and to ensure the privacy of the interviews and urine-drug screen.

What was measured for the stated domains? There is need to explain the scale applied and to describe how these were developed. Scaling and scoring of constructs including number of items operationalizing each domain,

Answer: to address this guidance on “explaining the scale applied and to describe how these were developed”, we added the following explanations also under the “Instrument development and measures”.

- The WHOQOL-BREF instrument: an abbreviated version of the WHOQOL-100, which covered 26 questions in four domains: physical, psychological, social relationships and environment. Responses to these 26 questions were recorded on 5-point Likert scale and transformed each raw scale score to a 0-100 scale using the formula.
- These are the steps for checking and cleaning data and computing domain scores for the WHOQOL-BREF.
- Step 1: Check all 26 items from the assessment having a range of 1-5
- Step 2: Convert 3 negatively phrased items into relevant positively framed questions.
- Step 3: Compute domain scores from relevant domain questions
- Step 4: Transform scores to a 0-100 scale

This step involves transforming each raw scale score to a 0-100 scale using the formula shown below: $Transformed Scale=[\frac{(Actual raw score-lowest possible raw score)}{Possible raw score range}$] × 100

- where “Actual raw score” is the value achieved through summation, and “lowest possible raw score” is the lowest possible value that could occur through summation. “Possible raw score range” is the difference between the maximum possible raw score and the lowest possible raw score (WHO, WHOQOL-BREF, 1997). These calculated QOL scores according to different domains and in-total scores were analysed with response characteristics to identify the association with QOL scores.”
- The statistical analysis appear casual for such a study demonstrating rigour of design and elucidation of dynamics.
- Measures using the Likert response scale without adjusting for measurement error inherent in transforming  responses from frequency distribution to scale development scores to give summaries of descriptive statistics of means and standard deviation has serious consequences of accuracy.

Answer: Thanks for pointing out the Likert response scale adjustment. Actually, we applied in analysis and hereby re-mention its steps of analysis as mentioned above steps.

- Step 2: Convert 3 negatively phrased items into relevant positively frame questions. (Detailed step by step explanation are mentioned above).
- The result under the WHO QOL scores of methadone patients describing quality of life scores showed serious flaw in the data analysis from the 5-response Likert scale demonstrating error in the use of a 1 to 5 scoring modality instead of zeroing the scale.

Answer: Thanks for highlighting the need of explanation of the analysis steps. As the WHOQOL-BREF is a validated questionnaires which is using across the world and we omitted its routine calculation procedures. However, I hereby now added the steps for its calculation method and reference for its full analysis. (Detailed step by step explanation are mentioned above).

- The sub-section of “Factors associated with quality of life (QOL) total score” has serious issues with weak statistical analysis expressing findings from the study data. What is communicated is inappropriate for expressing weighted-aggregate scores for quality of life measure
- Answer: More comprehensive explanation was added to have clear assumption on the analysis of the session “WHO QOL scores of methadone patients” as follows. “The patients' responses on their quality of life were rated with the 1-5 Likert scale. If the average rating was higher than 3 in all 26 QOL questionnaires, the respondent's total response average would be above 79. However, there can be far higher or lower responses than the middle rating of 3. In this analysis, the total of the average rating higher than 79 was calculated as a higher QOL total score. The higher WHO-QOL scores represent better quality of life and lower scores reflect poor QOL.”
- When we analyze the QOL total score of the individual respondents, we directly refer the total of the individual domain response rather than use of weighted score to avoid the misclassification of the data due to weighted calculation. So, in this case, we can have less classification due to direct use of response into 2 groups for identification of association.

==============================

Please submit your revised manuscript by Apr 21 2022 11:59PM. If you will need more time than this to complete your revisions, please reply to this message or contact the journal office at [globalpubhealth@plos.org](mailto:globalpubhealth@plos.org). When you're ready to submit your revision, log on to <https://www.editorialmanager.com/pgph/> and select the 'Submissions Needing Revision' folder to locate your manuscript file.

Please include the following items when submitting your revised manuscript:

- A rebuttal letter that responds to each point raised by the editor and reviewer(s). You should upload this letter as a separate file labeled 'Response to Reviewers'.
- A marked-up copy of your manuscript that highlights changes made to the original version. You should upload this as a separate file labeled 'Revised Manuscript with Track Changes'.
- An unmarked version of your revised paper without tracked changes. You should upload this as a separate file labeled 'Manuscript'.

Guidelines for resubmitting your figure files are available below the reviewer comments at the end of this letter.

We look forward to receiving your revised manuscript.

Kind regards,

Nnodimele Onuigbo Atulomah, PhD
Academic Editor
PLOS Global Public Health

Journal Requirements:

1. Please include a complete copy of PLOS’ questionnaire on inclusivity in global research in your revised manuscript. Our policy for research in this area aims to improve transparency in the reporting of research performed outside of researchers’ own country or community. The policy applies to researchers who have travelled to a different country to conduct research, research with Indigenous populations or their lands, and research on cultural artefacts. The questionnaire can also be requested at the journal’s discretion for any other submissions, even if these conditions are not met.  Please find more information on the policy and a link to download a blank copy of the questionnaire here: <https://journals.plos.org/globalpublichealth/s/best-practices-in-research-reporting#loc-inclusivity-in-global-research>. Please upload a completed version of your questionnaire as Supporting Information when you resubmit your manuscript.

Answer: There is a supplementary file answering PLOS’ questionnaire on inclusivity in global research.

2. Please update the completed 'Competing Interests' statement, including any COIs declared by your co-authors. If you have no competing interests to declare, please state "The authors have declared that no competing interests exist"

Answer: We updated completed 'Competing Interests' statement in the manuscript.

3. We see that your study includes live participants, but you have not included an Ethics Statement. Please update your manuscript file to include an Ethics Statement subsection to your Materials and Methods section. It should include:

iii) (for human participants or donors) - A statement that formal consent was obtained (must state whether verbal/written) OR the reason consent was not obtained (e.g. anonymity)

Answer: Consent related explanation was under the “Ethical Measures” session as follows.

“As the participants were the most-at-risk population and vulnerable, the research team requested a waiver of the local ethical review committee from participant signing, and it was granted for verbal consent procedure. The interviewer read the informed consent form for the participant and noted the obtained individual verbal consent. No particular identification was collected. All survey data and biological samples were analysed anonymously.”

4. In the online submission form, you indicated that "The [.dta] data used to support the findings of this study are available from the corresponding author upon the approval of the Centre for Drug Research.". All PLOS journals now require all data underlying the findings described in their manuscript to be freely available to other researchers, either 1. In a public repository, 2. Within the manuscript itself, or 3. Uploaded as supplementary information.
This policy applies to all data except where public deposition would breach compliance with the protocol approved by your research ethics board. If your data cannot be made publicly available for ethical or legal reasons (e.g., public availability would compromise patient privacy), please explain your reasons by return email and your exemption request will be escalated to the editor for approval. Your exemption request will be handled independently and will not hold up the peer review process, but will need to be resolved should your manuscript be accepted for publication. One of the Editorial team will then be in touch if there are any issues.

Answer: The [.dta] data used to support the findings of this study was included in the manuscript.

**Data:** The data used to support the findings of this study is available at [10.6084/m9.figshare.19663050](https://doi.org/10.6084/m9.figshare.19663050).

5. If you did not receive any funding for this study, please simply state: “The authors received no specific funding for this work.”

Answer: We added as ““The authors received no specific funding for this work.” In the manuscript.

Additional Editor Comments (if provided):

Two reviewers assessed the quality of the study reported in this manuscript submitted for possible publication which sort to explore Factors associated with quality-of-life scores among methadone patients in Myanmar and to elucidate the dynamics involved in the problem phenomenon related to treatment modality of those recovering from the use of potentially deadly chemical substances. The reviewers felt that revision of observed weaknesses in the manuscript is corrected would make it acceptable for possible publication. I strongly recommend that the authors attend to the areas pointed out as requiring modifications in the manuscript.


Answer: All the suggested points are addressed accordingly in the revised manuscript.

I also revised the “limitation” under the “Discussion” session with the additional sentence as “With the limitation of this study context, there were a few biases expected in this study. Selection bias from the specified selection criteria, observation bias as the Hawthorne Effect introduced by the service delivery organisations in conducting the survey, and recall bias from the respondents in answering the questions related to feeling and action during the specified timeline in the questions were observed.”

Reviewers' comments:

Reviewer's Responses to Questions

**Comments to the Author**

1. Does this manuscript meet PLOS Global Public Health’s [publication criteria](https://journals.plos.org/globalpublichealth/s/criteria-for-publication)? Is the manuscript technically sound, and do the data support the conclusions? The manuscript must describe methodologically and ethically rigorous research with conclusions that are appropriately drawn based on the data presented.

Reviewer #1: Yes

Reviewer #2: Partly

2. Has the statistical analysis been performed appropriately and rigorously?

Reviewer #1: Yes

Reviewer #2: Yes

3. Have the authors made all data underlying the findings in their manuscript fully available (please refer to the Data Availability Statement at the start of the manuscript PDF file)?

The [PLOS Data policy](https://journals.plos.org/globalpublichealth/s/data-availability) requires authors to make all data underlying the findings described in their manuscript fully available without restriction, with rare exception. The data should be provided as part of the manuscript or its supporting information, or deposited to a public repository. For example, in addition to summary statistics, the data points behind means, medians and variance measures should be available. If there are restrictions on publicly sharing data—e.g. participant privacy or use of data from a third party—those must be specified.

Reviewer #1: Yes

Reviewer #2: No

4. Is the manuscript presented in an intelligible fashion and written in standard English?

PLOS Global Public Health does not copyedit accepted manuscripts, so the language in submitted articles must be clear, correct, and unambiguous. Any typographical or grammatical errors should be corrected at revision, so please note any specific errors here.

Reviewer #1: Yes

Reviewer #2: No

5. Review Comments to the Author

Please use the space provided to explain your answers to the questions above. You may also include additional comments for the author, including concerns about dual publication, research ethics, or publication ethics. (Please upload your review as an attachment if it exceeds 20,000 characters)

Reviewer #1: Reviewers Comment:
PGPH-D-21-01098
Factors affecting the quality of life (QOL) scores among methadone patients in Myanmar

Overall, the authors have done well. However, these issues should be addressed to enhance the quality of the stiudy:
1. The concluding part of the abstract mainly highlighted the aim of the study. It should point out the factor that affected the quality of life in the study. Lines 25 and 25

Answer: We hereby revise in the conclusion of the abstract as “This study identified factors associated with QOL; infections, frequency of drug use and episodes of methadone treatments, satisfaction status on their leisure, and addressing these factors will improve the service intervention and the quality of life among methadone patients.”

2. The objective of the study ahoul end the introduction. Line 60

- As far as we understand, we hereby revise the last sentence at the end of the introduction session as “This study identified the associated characteristics of the respondents with the quality of life of the methadone clients. It also determined how these characteristics had differed the QOL scores. This analysis was the first of such kind of WHOQOL-BREF study for methadone patients in Myanmar.
- Those patients who took methadone at least 6 months in the methadone program were defined as retained clients by the DDTRU. As the patients in the MMT (Methadone Maintenance Treatment) were taking the methadone for a minimum of 6 months, this study aimed to identify the reported QOL and how QOL differed from the reported characteristics of methadone treatment by having at least a considerable duration.”

3. The result was poorly discussed. It should be free from the results there in. Instead, the results need to be interpreted considering the result here. There should be comparison, contrast, and possible description of the meaning. Summarize key results with reference to the study objectives

- Answer: Yes, We are following the guidance and improved the objectives in the abstract as
- “This study evaluated the factors associated with the quality of life (QOL) of methadone patients to ensure efficient service delivery. It also identified how patients’ characteristics had differed QOL scores of respondents.”

Further modification were also integrated into the result session.

- Under the “Factors associated with quality of life (QOL) total score”, the following text were added. “The relationship between respondents’ QOL and characteristics of methadone treatment was also evaluated.”

…..

- “This showed that those respondents who suffered from infectious diseases related to drug use and blood-borne infections related to injection, who were still on drug use and affecting the psychological response without having the satisfied leisure statuses had significantly associated with QOL issue. The data was further analysed t-test for two independent samples for the differences in the quality of life (QOL) total score against the respondents’ characteristics in table 2. Then, these findings showed clearly which characteristics of the respondents made higher or lower QOL total scores.”
- For the “comparison and contrast” of the associated factors, it was mentioned in the “Discussion” session with different country experience and comparison.
  - “Clients infected with HIV affected their QOL improvement, mainly in psychological and social relationships than physical domain (Fei, Yee, Habil, & Danaee, 2016). …………….
  - However, cohort findings proved an improvement in QOL score with the higher dose of methadone and longer duration of treatment (Wang, et al., 2012).…………
  - employment effects on increased QOL (Talebi, Zavar, Ghafari, & Poorandy, 2017), (Nguyen, et al., 2017). The unemployment lowered physical, psychological, environmental and social health of WHO-QOL score (Lin, Wu, & Detels, 2011). Those clients receiving income from temporary jobs and other non-criminal sources were significantly associated with the worse health-related QOL (HRQOL) scores in all domains (Yen, Chou, Lin, & Deng, 2015)……
  - Social network, and the relationship to family and friends were significant predictors and high QOL leisure time was associated with high overall QOL (Carlsen, Lunde, & Torsheim, 2019).”

4. How were the missing data addressed?

Answer: We hereby added the calculation steps related to the incomplete entries and missing data under the “Statistical Analysis” of the “Methods” session. “The analysis process omitted the incomplete entries in the calculation and missing data were not counted in the formula calculation to have a reliable estimate and reflected the actual QOL status of the respondents.”

5. Clearly identify and define all the outcomes for the study

Answer: We hereby added the outcomes at the end of the “Introduction” to have better idea outcomes for the study.

“This study identified the associated characteristics of the respondents with the quality of life of the methadone clients. It also determined how these characteristics had differed the QOL scores. This analysis was the first of such kind of WHOQOL-BREF study for methadone patients in Myanmar.

Those patients who took methadone at least 6 months in the methadone program were defined as retained clients by the DDTRU. As the patients in the MMT (Methadone Maintenance Treatment) were taking the methadone for a minimum of 6 months, this study aimed to identify the reported QOL and how QOL differed from the reported characteristics of methadone treatment by having at least a considerable duration.”

6. The paragraphs of the discussion section are rather too small and should be restructured

Answer: Discussion session was modified with the new additional supportive research findings from other country experience.

The structure of the paragraphs are combined with the relevant discussion points.

7. The eligibility criteria should be clearly defined and unambiguous. Apart from patients above 18 years old who gave their informed consent to participate in the study, what other factors was considered in the selection?

Answer: The eligibility criteria was modified in the manuscript as per the guidance.

The inclusion criteria was added as “The inclusion criteria specified those respondents who were at least 6-month treatment continuously in the recent enrolment on methadone treatment. However, there was no restriction on the first time of methadone treatment or additional episodes of the methadone treatment.”

8. What effort was made by the authors to address potential sources of bias in the study?

Answer: Potential sources of biases were added in the limitation of the “Discussion” session.

“With the limitation of this study context, there were a few biases expected in this study. Selection bias from the specified selection criteria, observation bias as the Hawthorne Effect introduced by the service delivery organisations in conducting the survey, and recall bias from the respondents in answering the questions related to feeling and action during the specified timeline in the questions were observed.”

9. The study limitations should be stated in line with sources of potential bias

The potential bias were added in the limitation session.

10. Most of the references are either incomplete or wrongly written. Some internet references do not have web address while those with web address do not have retrieval dates. All these should be tidied up.

Answer: Yes, the historic access of web links were restored and access of the websites were checked and added. Exception to some national programme reports which were not published online.

Additional 6 references relating to the modification of the manuscript was added and highlighted.

11. Correct all typographical and sentence errors.

Thank you. Reviewed and addressed all the incomplete sentences errors.

Reviewer #2: Overall, the topic is an interesting one and very informative .It is applicable to developed and developing countries alike. The authors however need to read through the manuscript and ensure that there is logical flow in the construction of sentences. The introduction in the abstract does not align with the objectives of the study and this should be corrected. The methodology needs to be revisited including clear description of how individual respondents were selected, details or reference to a protocol for urine testing for ilicit drugs, ethical considerations etc.
Other comments to the author are presented in the attached word document .

Answer: One sentence in the “Background” of the abstract was removed for the smooth flow about the methadone program and added the relevant info on QOL as “The quality of life (QOL) among the clients retained in the methadone program is important for their personal development and an indication of the treatment efficacy.”

- Method: Info on selection of respondents was added as

“From the eligible candidate list of the service providers/ resource people of the drop-in-centres, the respondents were recruited randomly and explained to them the objectives and benefits of the study. As the interested community was vulnerable and marginalised, respondents were recruited for participation who gave consent to participate in the survey interview and those who agreed to provide urine specimens linked with their response. Individual interview process and urine sample collection were done at the drop-in-centres or private rooms of the restaurants for the privacy of the assessment.”

- The logical flow was checked and added the additional sentences and editing the phrases throughout of the manuscript for the smoothness and the logical flow of the manuscript.

6. PLOS authors have the option to publish the peer review history of their article ([what does this mean?](https://journals.plos.org/globalpublichealth/s/editorial-and-peer-review-process#loc-peer-review-history)). If published, this will include your full peer review and any attached files.


**Do you want your identity to be public for this peer review?** If you choose “no”, your identity will remain anonymous but your review may still be made public.


For information about this choice, including consent withdrawal, please see our [Privacy Policy](https://www.plos.org/privacy-policy).

Reviewer #1: **Yes:** Ogbonna Brian Onyebuchi

Reviewer #2: No

[NOTE: If reviewer comments were submitted as an attachment file, they will be attached to this email and accessible via the submission site. Please log into your account, locate the manuscript record, and check for the action link "View Attachments". If this link does not appear, there are no attachment files.]

While revising your submission, please upload your figure files to the Preflight Analysis and Conversion Engine (PACE) digital diagnostic tool, <https://pacev2.apexcovantage.com/>. PACE helps ensure that figures meet PLOS requirements. To use PACE, you must first register as a user. Registration is free. Then, login and navigate to the UPLOAD tab, where you will find detailed instructions on how to use the tool. If you encounter any issues or have any questions when using PACE, please email PLOS at [figures@plos.org](mailto:figures@plos.org). Please note that Supporting Information files do not need this step.
